# A novel electroporation system for efficient molecular delivery into *Chlamydomonas reinhardtii* with a 3-dimensional microelectrode

**DOI:** 10.1038/srep15835

**Published:** 2015-11-02

**Authors:** Seongsu Kang, Kwon-Ho Kim, Yeu-Chun Kim

**Affiliations:** 1 Korea Advanced Institute of Science and Technology (KAIST), Department of Chemical and Biomolecular Engineering, Daejeon, 305-701, Republic of Korea

## Abstract

Electroporation is one of the most widely used transfection methods because of its high efficiency and convenience among the various transfection methods. Previous micro-electroporation systems have some drawbacks such as limitations in height and design, time-consuming and an expensive fabrication process due to technical constraints. This study fabricates a three dimensional microelectrode using the 3D printing technique. The interdigitated microstructure consisting of poly lactic acid was injected by a 3D printer and coated with silver and aluminum with a series of dip-coatings. With the same strength of electric field (V cm^−1^), a higher efficiency for molecular delivery and a higher cellular viability are achieved with the microelectrode than with a standard cuvette. In addition, this study investigates chemicophysical changes such as Joule heating and dissolved metal during electroporation and showed the micro-electroporation system had less chemicophysical changes. It was concluded that the proposed micro-electroporation system will contribute to genetic engineering as a promising delivery tool, and this combination of 3D printing and electroporation has many potential applications for diverse designs or systems.

Introducing foreign molecules such as genes and proteins into cells has been regarded as a very significant technique in various biological studies. Delivering exogenous nucleic acids into eukaryotic cells, a process known as transfection, has enabled a wide range of applications including gene therapy^1^,^2^, DNA vaccines^3^,^4^, *in vitro* fertilization^5^, cancer treatment^6^,^7^, tissue engineering^8^, induced pluripotent stem (iPS) cell^9^,^10^ and metabolic engineering^11^. Because cell membranes are not permeable to most foreign macromolecules, particular methods are necessary for delivering molecules into cells. Among the various methods including electroporation^4^,^12^, glass beads^13^, viral vector^14^, chemical treatment^9^, and bombardment^15^,^16^, electroporation is one of the most widely used methods for the transfection of cells because of its fast delivery, technical simplicity, and applicability to diverse cell types and sizes.

However, the conventional cuvette type electroporation system requires high voltage and undergoes metal ion dissolution^17^,^18^, excess heat generation^17^,^19^, local pH variation^17^,^18^, electric field distortion^17^, and irreversible electroporation of the transmembrane caused by excessive voltage, resulting in low efficiency of electroporation and low cell viability. According to previous research, it is believed that higher cell viability and transfection efficiency can be achieved by narrowing the distance between electrodes by solving the problems presented by a conventional cuvette type electrode^17^,^19^,^20^. This reduction in the distance between electrodes means a lower voltage can be applied to reach the same electric field strength (V cm^−1^) and has become one of the main concepts of micro-electroporation systems in the last decade.

Many types of micro-electroporation systems including microelectrode, microchannel and microcapillary systems have been developed. Most of the fabrication methods for these micro-electroporation systems are based on micro electro mechanical system (MEMS) technology, which limits the electrode’s height to ~0.1 mm. A technical constraint of MEMS is that only a small confined region can be electroporated in practice, which means that only small volumes of samples can be electroporated at one time^21^,^22^. The issue of high cost is also considered as one of the problems with the MEMS process because it commonly involves expensive procedures such as lithography, metal deposition, etc^23^,^24^,^25^. Additionally, in a microchannel system, expensive equipment and channel blocking by cells or bubbles from electrolysis have been big issues with the system^26^,^27^,^28^,^29^.

In this study, we developed a novel micro-electroporation system using a three dimensional microelectrode fabricated by 3D printing. 3D printing technology has been spotlighted by creating bespoke, low-cost appliances with rapid prototyping and patterning^30^. It has been utilized in the casting field and in bone engineering mainly. However, recently, it also has been used in diverse fields such as catalytic chemistry^31^,^32^, bioceramics^33^, tissue engineering^34^, microfluidic devices^35^ and electro-devices^36^.

We fabricated a 3 dimensional PLA (poly lactic acid) structure with 3D printing and coated the structure with metal using a dip-coating method. Fabrication based on 3D printing ensures a time-saving, easy, simple and economical process compared to previous fabrication processes such as MEMS^31^. Using the novel 3-dimensional micro-electroporation system, *Chlamydomonas reinhardtii*, the standard model strain for microalgae was electroporated to investigate the delivery efficiency and cell viability. Microalgae has received significant attention as a bioresource for the production of biodiesel because it accumulates lipids in their cell bodies comprising between 20% and 50% of their cell body^37^. For practical use in biodiesel production, enhancing the algal biology such as photosynthetic efficiency or oil contents using genetic engineering has been studied by many researchers. Although the transfection of algal cells with exogenous genes is inevitable work in this step, quite a low transfection efficiency compared to other cell lines such as animal cells is still the main hurdle in algal transfection studies.

By introducing an unprecedented 3D microelectrode system fabricated by 3D printing, higher cellular viability and intracellular delivery efficiency for several kinds of molecules in algal cells were achieved, compared to the conventional cuvette system. Furthermore, our micro-electroporation system has superior characteristics such as decreased Joule heating and a lower concentration of metal compared to a conventional system both of which are known to be harmful factors to cells. Our micro-electroporation system will improve the transfection efficacy of cells and greatly contribute to genetic engineering as a promising gene delivery tool.

## Results

### Intracellular delivery of tracer molecules

**Figure 1:**
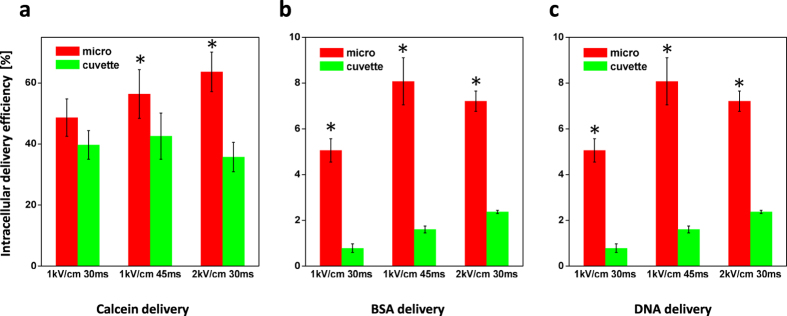
Intracellular delivery efficiency measured by flow cytometry. (**a**) Calcein (660 Da) (**b**) FITC-BSA (66 kDa) (**c**) Plasmid DNA (4 kb). Data represent the average of n = 3 replicate experiments. Standard deviation bars are shown. *Significantly different (Student’s *t*-test, p < 0.05).

**Figure 2:**
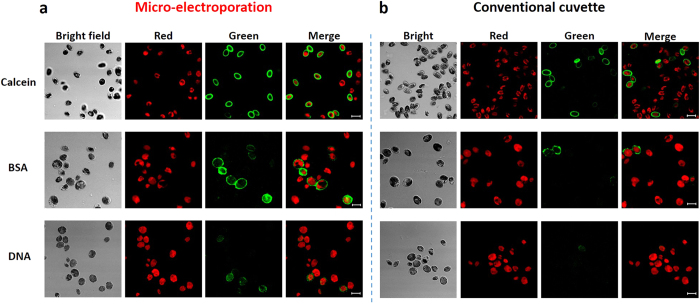
The comparison of confocal microscopy images after intracellular uptake of tracer molecules in cell-wall-deficient mutant *C. reinhardtii* through electroporation using (a) microstructure, (b) commercialized cuvette when 1 kV cm^−1^ and 45 ms pulse was applied. The red color indicates the chlorophyll of algal cell. Scale bar means 10 μm.

Electroporation with the 3D micro-electroporation system and a conventional cuvette system was done with 3 kinds of tracer molecules: calcein, FITC-BSA and plasmid DNA. The number of pulses was fixed at one time, and the pulse voltage and duration were varied. The optimal electrical parameters were determined by fluorescence intensity with photoluminescence spectroscopy and viability based on the quantum yield (Fig. S1). Using various electrical parameters, our micro-electroporation system showed improved delivery efficiency compared to the conventional cuvette system (Figs 1 and 2). In the case of the calcein and FITC-BSA, the delivery efficiencies were increased by approximately 1.5-fold and 7-fold, respectively. For intracellular delivery of plasmid DNA, the delivery efficiency increased by approximately 4.85-fold using the microelectrode. For calcein, the delivery efficiency in the electroporated cells by cuvette decreased when the strength of the electric field increased from 1 kV cm^−1^ to 2 kV cm^−1^, which was in agreement with a previous study on the electroporation of *Chlamydomonas* showing the same decreasing trend^38^.

Considering the relationship between the size of the tracer molecule and the delivery efficiency, it seems that the micro-electroporation system forms larger sizes of pores that emerge when the electrical pulse is applied and is more suitable for delivering macromolecules into cells than that of a conventional cuvette. To verify this hypothesis regarding the pore size, more studies on membrane modeling and *in-situ* observations of pore formation are necessary. As shown in Fig. 1, the trends in the graphs when the electrical parameters changed were different between the micro-electroporation and cuvette system. It seems that the optimal electrical parameters for the microelectrode and cuvette are slightly different. It appears that the microelectrode has a higher delivery efficiency and overcomes the efficiency threshold of cuvette type electroporation by modulating the electroporation parameters.

Collectively, we conclude that our novel micro-electroporation system is a more appropriate option for the delivery of not only small molecules such as calcein but also macromolecules like proteins and DNA and can greatly contribute to the advancement of basic biological science.

### Cellular viability

Electroporation causes both reversible and irreversible nano-size pores. Irreversible pores in which transiently formed pores cannot recover are quite fatal to cells causing cellular death. It is well known that the cellular viability is as important as the delivery efficiency in electroporation because cell viability is directly related to the final number of transformants. Therefore, there have been many studies on improving cellular viability in electroporation or on optimizing the electrical parameters^12^,^39^.

**Figure 3:**
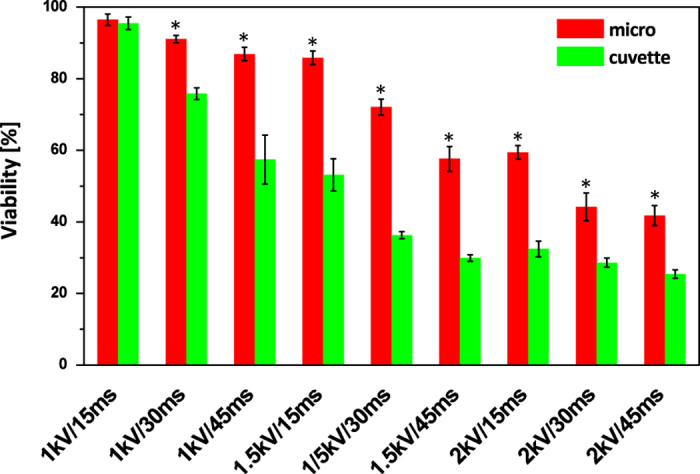
The viability of algae cells based on quantum yield. Normalized viability was calculated by dividing the quantum yield (QY) of the control group by the QY of the experimental group. Data represent the average of n = 5 replicate experiments. Standard deviation bars are shown. *Significantly different (Student’s *t*-test, p < 00.05).

To investigate the cellular viability of electroporated algal cells, the quantum yield was measured which indicates the ability of photosynthesis system II. In nine different electrical parameters, micro-electroporation had superior cellular viability (Fig. 3). For three of the electrical parameters mentioned in the section on the intracellular delivery efficiency, the viabilities of the micro-electroporated cells were 91%, 86% and 72% for 1 kV cm^−1^ at 30 ms, 1 kV cm^−1^ at 45 ms, and 2 kV cm^−1^ at 30 ms, respectively; otherwise, the viabilities of the electroporated cells with a conventional cuvette were 75%, 57% and 36% for the 3 parameters mentioned above, respectively.

Some sensitive cell lines such as stem and primary cells have showed a low transfection efficiency and viability with electroporation. Furthermore, for cell lines with intact cell walls, high voltages have to be applied for effective molecular delivery, resulting in low cell viability^12^,^38^. Our micro-electroporation system will contribute to the transfection studies of these hard-to-transfect cells by solving the low cellular viability problem.

### Dissolved Al ion from the electrode

Among various kinds of metal, aluminum has been widely used as an electrode material for electroporation because of its high conductivity, ease of manufacturing and low cost. Previous research revealed that dissolved aluminum and aluminum ions were generated at both the anode and cathode after electroporation^18^,^40^.

Anode:


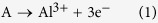


Cathode:


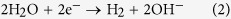



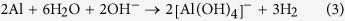


In solution:


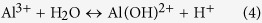



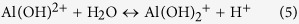



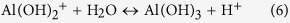


**Figure 4:**
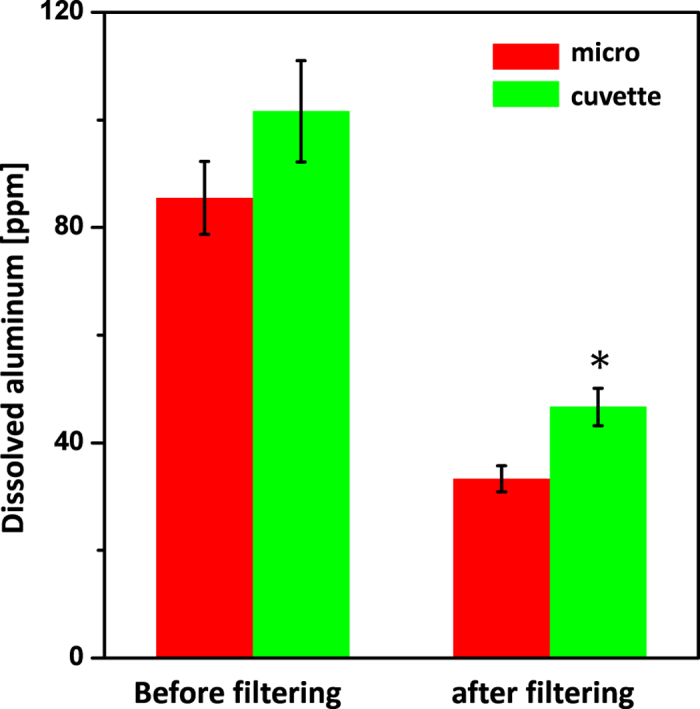
Dissolved Al concentration in buffer (PBS) after electroporation analyzed by ICP-MS. The both concentrations of dissolved aluminum of filtered buffer and non-filtered buffer were measured. Data represent the average of n = 3 replicate experiments. Standard deviation bars are shown. *Significantly different (Student’s *t*-test, p < 0.05).

The generated aluminum ions and aluminum oxides are important factors in electroporation because they can cause toxic effects and a conglomeration of cells and aluminum complexes^18^,^41^. After electroporation, some flocculated precipitates were formed and observed by the naked eye. This observation was in agreement with previous research. The both concentrations of dissolved aluminum in the electroporated PBS buffer containing precipitates and the filtered buffer were measured by inductively coupled plasma mass spectrometer (ICP-MS) (Fig. 4). With the same electrical parameters (1 kV cm^−1^ for 45 ms), a lower concentration of dissolved aluminum was detected when using the microelectrode than when using a conventional cuvette. Aluminum complexes originating from the electrodes could conglomerate with cellular debris and media constituents which has been actually observed under a microscope. According to previous research, it was revealed that cluster formation dramatically reduces the specific proliferation rate, and shear forces exerted on the aggregates cause a considerable higher specific death rate than those exerted on a single cell^42^. In the case of microalgae for which the ability of photosynthesis is quite an important factor for cell growth and proliferation, cell aggregation could also inhibit the photosynthesis efficiency of individual cells by decreasing the effective area of the cell surface which absorbs photons. The lower concentration of aluminum detected in the micro-electroporation system could be one of the reasons why the micro-electroporation system has higher cellular viability than that of a standard cuvette. Furthermore, considering that electroporated and damaged algal cells are fragile when incubated in transfection studies, the micro-electroporation system seems to be better choice for future algal transfection studies.

### The ability of retention of dye in the cellular body

After the cells were electroporated with the tracer dyes calcein and FITC-BSA, the intracellularly delivered dyes tended to “leak” out. The fluorescent intensity of the media from the samples increased over time while the fluorescent intensity of the electroporated cells decreased. For more damaged cells electroporated with harsh electrical parameters, this decaying fluorescent intensity was prominent. How many external molecules cells can retain also seems to be related to the intactness of the cellular membrane. This has also been directly linked to successful studies on intracellular delivery of proteins and DNA. The target molecules act on the cells for longer periods because the target molecules are retained for a longer time.

**Figure 5:**
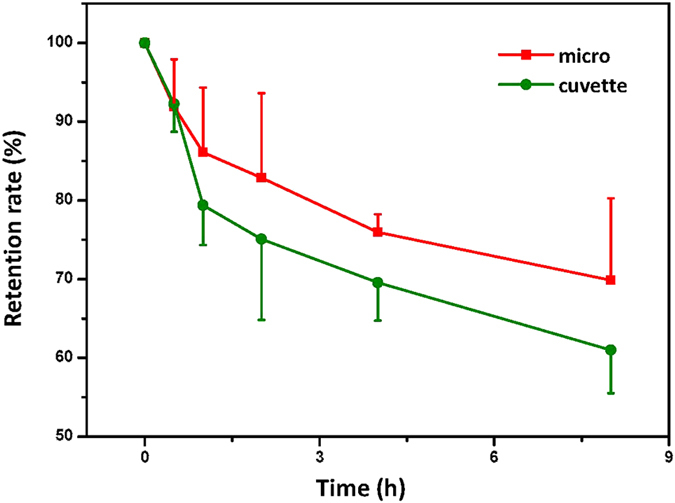
Retention rates of fluorescent molecule (FITC-BSA) uptaken into algal cells which were electroporated by cuvette and microelectrode. The 1 kV cm^−1^ and 45 ms pulse was applied. Data represent the average of n = 3 replicate experiments. Standard deviation bars are shown.

The retention rate of the tracer molecule FITC-BSA in the cells was measured with a photoluminescence spectrometer over time. The retention rate (%) is calculated by dividing the fluorescence intensity at specific time by the initial fluorescence intensity. Compared to the sample electroporated with a cuvette, a higher retention rate of fluorescent molecules in cells was achieved with the microelectrode (Fig. 5). After one hour, cells electroporated with the microelectrode had about a 5 percent higher retention rate than the cells electroporated with the cuvette type. As time had gone, the difference of retention rate between samples which were electroporated with microelectrode and cuvette tended to be increased. Finally, about a 10 percent higher retention rate was observed in the cells electroporated with the microelectrode after 8 hours. A higher retention rate is also expected to be observed for DNA delivery in the same manner. In DNA transfection studies, the possibility of plasmid DNA integrating into the genome could increase when the target DNA stays for a long time in the cytosol. Our micro-electroporation system will have a prominent role as an effective intracellular delivery tool for basic science research.

### Joule heating generated during electroporation

**Figure 6:**
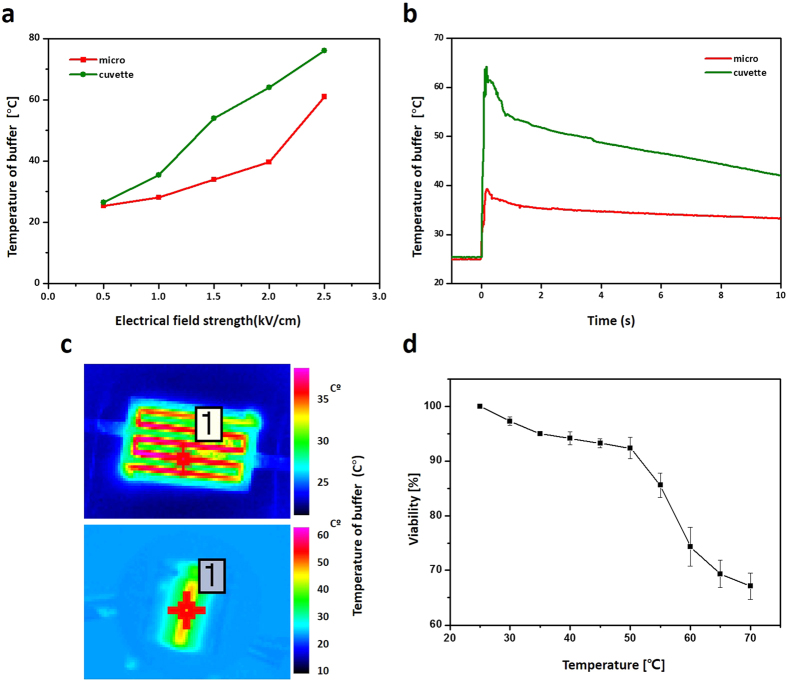
Joule heating analyzed by thermal infrared camera. (**a**) The maximum elevated temperature of electroporation buffer (TAP) in microelectrode and cuvette and (**b**) The temperature changes in real time when 2 kV cm^−1^ and 30 ms pulse was applied. (**c**) The infrared camera image when 2 kV cm^−1^ and 30 ms was applied. (**d**) The cell viability according to the temperature measured immediately after suspension. Resuspension and measurement were carried out in at least one second.

When an electrical pulse was applied to the buffer between the two counter electrodes, the temperature of the buffer increased. This Joule heating phenomenon has been regarded as an important factor in electroporation due to its detrimental effect although this is debatable^17^,^19^,^43^. Previous studies have focused on joule heating in electroporation through simulated 2D or 3D modeling^43^,^44^,^45^. Although there have been reported cases for measuring the increased buffer temperature via thermocouple under markedly long pulses (~2 second), not applied for actual transfection^46^ and the increased buffer temperature in continuous flow of microfluidic device^47^, no one has measured elevated temperatures of the electroporation buffer at “the moment” that is the electrical conditions of ~100 ms which is used in *in-vitro* reversible electroporation. Furthermore, whether radiated heat has adverse effects on cells has not been shown clearly yet because of the lack of a method for estimating cell viability in real-time. Here, we measured the actual heat generated when electroporation was applied using an infrared thermal camera. Comparing the previous method using thermocouple, the infrared thermal camera showed a shorter response time while making it possible to detect the changes of temperature within few milliseconds. With the various electric field strength parameters, lower temperature was detected with the microelectrode (Fig. 6). The majority of the heat, about 90%, was maintained in the initial, first second and gradually dissipated thereafter. It was observed that the elevated temperature was maintained over 10 seconds. In the delivery of proteins, elevated temperatures can cause structural deformation and denaturation which can lead to the protein losing its functionality. In the same manner, DNA will also undergo denaturation in highly elevated temperatures, resulting in a reduced efficiency of transfection.

To determine the correlation between the temperature of the buffer for electroporation and cellular viability, the viability of the algal cells by varying the temperature of the media was measured (see Fig. 6d). In this experiment, dark adapted cells were resuspended into heated media, and the quantum yields were measured rapidly. Comparing other cell viability assay such as PI staining or MTT assay which require long time, measuring quantum yield of algal cell immediately (~1 second) is possible and can be regarded as appropriate method for study of relationship between cell viability and elevated temperature after electroporation. The viability based on the quantum yield decreased when the temperature of the media increased. The decrease in viability seemed to be due to the denaturation of proteins such as biological enzymes. Based on that result, it can be said that elevated temperature by electrical pulse adversely affects the cellular viability in electroporation and should be strictly controlled. Collectively, our micro-electroporation system generated less heat which will decrease the thermal damage done to cells and the thermal deformation of target molecules. In addition, our system will enhance the electroporation performance for heat-sensitive cell lines such as specific cancer cells that easily suffer necrosis in temperatures ranging from 42 °C ~ 46 °C compared to normal cells.

## Discussion

In this study, we showed that the promising 3D printer technique can be applied to molecular biology studies and developed a highly effective micro-electroporation system for microalgae using a novel 3D printing method which is simple, economical and highly versatile for various designs. With the proposed microelectrode, higher intracellular delivery was achieved for various kinds of molecules including a florescent dye (calcein), protein (FITC-BSA) and plasmid DNA with optimized electrical parameters. Higher cellular viability was also achieved when using the proposed micro-electroporation system. Furthermore, we investigated the changes in the chemicophysical properties of the electroporation buffer such as Joule heating and aluminum concentration which can affect the cellular viability. Cells electroporated with the microelectrode retained cell impermeable protein molecules for a longer time than cells electroporated with a conventional cuvette suggesting that the micro-electroporation system results in longer effective times for target molecules such as proteins and DNA to function in cells.

A small quantity of sample in previous microstructures fabricated with MEMS, which should be solved for practical use, was also solved by introducing 3D printing to fabrication. This study also demonstrated the potential of 3D printing technique in electroporation by introducing a 3D printed structure for the electrodes and diverse operation system. In addition, this combination of the 3D printing technique and electroporation will make various complicated structures that have been considered impossible to manufacture or fabricate due to the technical constraints of previous fabrication methods possible for use in the electroporation field, leading to more efficient transfections. In addition, considering that a metal 3D printing technique, which extrudes metal material directly, was developed recently^48^,^49^,^50^, the possible designs for microelectrodes in our micro-electroporation system seems limitless. Our microelectrode fabricated by 3D printing can also be easily mass produced and will contribute to the commercialization of the micro-electroporation tool as a universal transfection tool, not just a theoretical tool in a study.

In other various studies such as proteomics, therapeutics and genetic engineering studies, the proposed micro-electroporation system fabricated by 3D printing is expected to play a remarkable part as an effective intracellular delivery tool for various kinds of proteins, and DNA and RNA molecules and serve as a basic design for various novel designs of micro-electroporation systems.

## Methods

### Fabrication of the three dimensional microelectrode device

**Figure 7:**
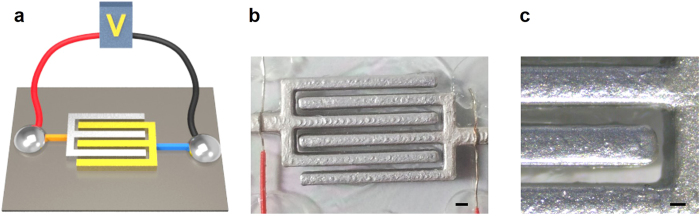
The fabricated microelectrode for micro-electroporation system. (**a**) The principle schematic diagram of micelectroporation system system (**b**) Actual image of microelectrode fabricated (scale bar: 1 mm) (**c**) Enlarged view of microelectrode (scale bar: 500 μm).

First, the halves of the interdigitated microscale patterns were designed with a computer-aided design program and then converted to a stereo-lithography (STL) file. Based on the STL file that included the design of the microelectrode, the G-codes were generated with the CreatorK program which is a utility program for operating the 3D printer. CreatorK was supplied by the manufacturer of the 3D printer, Rokit. Finally, the electrode structures were printed out by the 3D printer (3dison plus, Rokit, Korea) with a thickness of 1 mm, a height of 2 mm and a length of 1.8 mm. The printed structure consisting of poly lactic acid (PLA) was dipped into a diluted silver paste (Elcoat p-100, Cans, Japan) several times, and the silver-coated structure was serially dipped into an aluminum spray coating liquid (Loctite Al spray, Henkel, Korea). After sufficiently drying the microelectrode at room temperature, the two halves of the interdigitated microelectrode were arranged with tweezers under an optical microscope (Leica S6D, Leica Microsystem, German) and attached to glass with an adhesive. The distance between the interdigitated electrodes was 500 μm (Fig. 7). To conveniently connect the microelectrode and electrical pulse generator, wires were attached to each electrode. Our fabrication process is very simple (~5 min per one device) and low-cost (0.1 $ per one device) compared to other fabrication methods based on MEMS, which means better prospects for commercialization.

### Cell preparation

The microalgae *C. reinhardtii* CC-4348, which is a cell wall-less mutant, were grown in tris-acetate-phosphate (TAP) medium with continuous illumination, constant temperature (25 °C) and agitation (140 rpm). During incubation, the palmelloid state that an indefinite number of algae cells are embedded in a mucilaginous or gelatinous matrix was observed. The palmelloid state is induced by excessive metal ions or specific chemicals. Not only abnormal incubation conditions but also the type of cell line could affect the palmelloid state^51^. In the cellular uptake experiment, it was ambiguous and controversial whether the target molecules went into the cell or only into the palmelloid membrane which is located outside of the individual cells. At the exponential phase, the TAP medium was replaced with minimal media (Sager-Granick) which is known to eliminate palmelloid formation in laboratory cultures. One day later, the cells were harvested and resuspended in TAP media at a final concentration of 1.5 × 10^8^ cells ml^−1^ using a hemocytometer.

### Electroporation of the cell

Fluorescent tracer molecules (Calcein and FITC-BSA) were added to prepared cell solution at a final concentration of 100 μM and 10 μM, respectively. The Pyrexia tenera/Chlamydomonas reinhardtii CFP (pPtCrCFP) plasmid was supplied by Dr. Jeong at the Korea Research Institute of Bioscience and Biotechnology (KRIBB). The plasmid CFP (pCFP) was fluorescently labelled with a DNA intercalating dye, YOYO-1 at a ratio of 100:1, DNA base pairs (bp) to dye molecules. For homogeneous staining, the mixture or YOYO-1 and DNA was incubated for 2 hours at 50 °C in a dark room. The incubated DNA with YOYO-1 was added to the sample at a final concentration of 2 μg ml^−1^. Then, 80 μl of samples, which consist of tracer molecules and cells, were transferred to the micro-electroporation chip and conventional cuvette with a 2 mm gap (Model 620, BTX, USA). An electric field was applied by an electroporation pulse generator (ECM 830, BTX, USA). After applying the electric pulse, the mixture was transferred into a microcentrifuge tube with TAP media and incubated at 25 °C for 30 min for the recovery of the cells. Then, the cells were washed two times with TAP media using a centrifuge, and all the supernatants were discarded. After a second washing, the samples were treated with trypsin for 15 min at 37 °C to effectively eliminate fluorescent molecules bound to the cell membrane. After trypsin treatment, the samples were additionally washed with TAP media.

### Molecular uptake and viability

The intracellular molecular uptake efficiency was measured by a cell cytometer (MoFlo XDP, Beckman Coulter, USA). The cell was regarded as “fluorescing” if its fluorescence intensity was greater than the background signal from 99% of the untreated control cells. The fluorescence intensity of a bulk sample was measured by a spectrofluorophotometer (RF-5301 pc, Shimadzu, Japan) with an excitation wavelength of 488 nm when detecting calcein. The image for cellular uptake was taken by confocal laser scanning microscopy (FV1000 Live, Olympus, Japan) with an excitation wavelength of 488 nm.

Cell viability after electroporation was assayed by measuring the quantum yield (QY) of chlorophyll in photosynthetic unicellular eukaryotes. QY was measured by AquaPen-C APA100 (Czech Republic). The electroporated samples were incubated for one hour in the dark for dark adaptation. Normalized viability was calculated by dividing the QY of the experimental group by the QY of control group.

## Additional Information

**How to cite this article**: Kang, S. *et al.* A novel electroporation system for efficient molecular delivery into *Chlamydomonas reinhardtii* with a 3-dimensional microelectrode. *Sci. Rep.* **5**, 15835; doi: 10.1038/srep15835 (2015).

## Supplementary Material

Supplementary Information
