# Supplementary material for: A novel electroporation system for efficient molecular delivery into Chlamydomonas reinhardtii with a 3-dimensional microelectrode
Source: Sci Rep. 2015 Nov 2;5:15835. doi: 10.1038/srep15835 (PMC4629139; doi:10.1038/srep15835)
Supplement: Supplementary Information [file srep15835-s1.doc]

**Supplementary Information for Scientific Reports**

**A novel electroporation system for efficient molecular delivery into *Chlamydomonas reinhardtii* with a 3-dimensional microelectrode**

# Seongsu Kang, Kwon-Ho Kim, and Yeu-Chun Kim^+^

Korea Advanced Institute of Science and Technology (KAIST), Department of Chemical and Biomolecular Engineering, Daejeon, 305-701, Republic of Korea

^+^dohnanyi@kaist.ac.kr

**Supplementary Figure 1.** Optimization of electrical parameter for electroporation using photoluminescence spectroscopy and viability. A) Normalized fluorescent intensity of calcein uptaken in cells. B) the delivery efficiency calculated by multiplying normalized fluorescent intensity and normalized viability in **Fig 3**.
